# Impact of ACA implementation on health related quality of life among those with depressive disorders in the United States: A secondary data analysis of the 2011-2017 BRFSS

**DOI:** 10.1371/journal.pone.0266402

**Published:** 2022-03-31

**Authors:** Kathryn Mazurek, Wei Xue, Marissa Beldon

**Affiliations:** 1 Department of Health Sciences, Northern Illinois University, DeKalb, IL, United States of America; 2 Department of Biostatistics, University of Florida, Gainesville, FL, United States of America; IGMC: Indira Gandhi Medical College, INDIA

## Abstract

**Purpose:**

The passage of the Affordable Care Act in the US resulted in more Americans with health insurance coverage as well as expanded health benefits. However, barriers in accessing health care still exist in the US especially as it relates to some of the most vulnerable Americans including those with depressive disorders. The purpose of this cross-sectional secondary data analysis was to examine the differences in health-related quality of life for individuals with depressive disorders in early years of the implementation of the Affordable Care Act as compared to later years of implementation.

**Methods:**

This study used a repeated cross-sectional design that pooled data from the 2011–2017 Behavioral Risk Factor Surveillance System which is a nationally representative survey of the non-institutionalized U.S. population. Logistic regression models were used to evaluate the before and after impact of the Affordable Care Act on health related quality of life for those with depressive disorders.

**Results:**

Those with depressive disorders in early years of implementation of the Affordable Care Act were less likely to report 14 or more days of poor physical health (AOR  =  0.96; 95% CI: 0.95, 0.98), were less likely to report 14 or more days of poor mental health (AOR  =  0.93; 95% CI: 0.92, 0.94), and less likely to report 14 or more days of overall poor physical and mental health (AOR  =  0.93; 95% CI: 0.90, 0.96) as opposed to later years of implementation.

**Conclusions:**

Our results indicate poorer health related quality of life for those with depressive disorders in later years of implementation of the Affordable Care Act. Despite expanded mental health benefits under the Affordable Care Act, those benefits do not always translate into improved access or improved patient-reported outcomes. The federal government needs to comprehensively address mental health services in order to improve patient-reported outcomes and mental health treatment for those with depression.

## Introduction

The National Alliance on Mental Illness reports that 19.4 million people in the United States will experience a major depressive episode in 2019 [1]. Although a small number of people may only have one episode of a depressive disorder in their lifetime, most individuals experience depressive disorders (DD) as a chronic illness and have multiple depressive episodes [2]. Among adults, DD are the leading cause of disability in the U.S. and depression is implicated in more than 50% of suicide attempts [2–4]. Despite patients requiring ongoing care, depressive disorders are often untreated or under-treated [5]. Of those diagnosed, only 62% of adults with DD receive long term treatment while over a third of adults do not receive any treatment at all [1].

Twenty-million people have benefited from insurance expansion under the Affordable Care Act (ACA) [6]. The main objectives of the 2010 legislation were to expand health care coverage to more people, expand state Medicaid programs, and reduce costs associated with delivery of care through innovation [7]. In 2014, the ACA, through the Essential Benefits Package, included provisions to improve access to mental health care services and extended the Mental Health Parity and Addiction Equity Act [8, 9]. As a result, all marketplace health insurance plans were required to offer mental health services along with mental health benefits on parity with medical benefits [9]. Individual and small group insurance plans covered mental health services with no annual or lifetime financial limits for mental health benefits. Large group plans, although not required to offer the Essential Benefits Package, could opt-in to provide essential health benefits [9]. Insurers that opted-in were required to provide mental health coverage without monetary limits and on parity with medical and surgical benefits [9]. Additionally, by expanding state Medicaid programs, it allowed Medicaid, the largest payer of behavioral health nationwide, to enroll even more Americans and provide mental health coverage [10].

Despite the potential gains from legislative reform with the passage of the ACA, access to much needed mental health services continues to be expensive, inconsistent, and health service utilization is still a challenge for those with mental illness [11]. These barriers in accessing health care have been highlighted by the pandemic and adversely affect health-related quality of life (HRQoL) for those with DD [12]. Patient reported outcome measures, like HRQoL, are used to outline improvements in patient centered medical treatment and health care. Clinicians and public health experts have used HRQoL to measure the effects of chronic illness, treatments, and short- and long-term disabilities [13]. Measuring HRQoL at the population level has been shown to be sensitive enough to measure system level effects. Outcome measurements such as HRQoL are imperative in making any system of care accountable [14]. Therefore, the purpose of this pooled cross-sectional secondary data analysis is to examine the differences in health-related quality of life for individuals with depressive disorders in early years of ACA implementation compared to later years of implementation via a before and after analysis. The results will fill a gap in the literature regarding HRQoL for those with depressive disorders as it pertains to the ACA and will provide recommendations for improving the mental health care system.

## Methods

The Behavioral Risk Factor Surveillance System (BRFSS) is a population-based health surveillance system conducted by the CDC that collects information on U.S residents 18 years and older regarding individual health risk behaviors and prevention practices affecting health status [15]. Additionally, the BRFSS collects information on health care access and self-reported well-being [16]. The CDC makes decisions regarding “public health research, practice, and policies” based on the health data collected from BRFSS participants and provides information used to coordinate public health services at the local, state, and national levels [16]. Surveillance system such as this allow for the ability to determine and address disparities and unmet health care needs [17].

This study used a repeated cross-sectional design that pools data from the 2011–2017 BRFSS, a nationally representative survey of the non-institutionalized U.S. population which included all 50 states and US territories. Logistic regression models were used to evaluate the association between health-related quality of life and implementation of ACA. The analysis adjusted for sex, age, race, education, marital status, employment status, health care coverage and income level. Because changes were made to BRFSS procedures in 2011 regarding cell phones and raking, comparison of pre-2011 BRFSS data to post-2011 data was not possible. Although the ACA was passed into law in 2010, mental health provisions were not applied until 2014. Thus, for this study, 2011 to 2013 are considered early or pre-ACA implementation years and 2014 to 2017 are considered later or post-ACA implementation years.

DD was determined by a single question in the BRFSS. The BRFSS participants were asked whether a medical professional had ever told them they have a depressive disorder such as depression, major depression, dysthymia, or minor depression [18]. The respondents who self-reported that a medical professional had ever told them that they have a depressive disorder were included in the analysis [18].

The BRFSS evaluates HRQoL using the CDC’s Healthy Days Core Module (CDC HRQOL– 4) [19]. This instrument measures perceived health via questions about “general health, physical health, mental health, and overall physical and mental health including activity-limited days” [19]. This analysis had four models with each of the HRQoL questions serving as a dependent variable. General health status was entered into the model as: excellent, very good, good and fair, poor. Questions about physical health, mental health, and overall physical and mental health prompted participants to identify how many days in the last 30 days they experienced symptoms. Because clinicians identify 14 or more symptom days in a 30-day period as an indication of clinical depression, responses were dichotomized as 13 or fewer days of symptoms and 14 or more days of symptoms [20, 21].

There are many adjustor variables in the models used to isolate the effect of ACA policies on HRQoL. The age categories used in the analysis included 18–24 years old, 25–34 years old, 35–44 years old, 45–54 years old, 55–64 years old, and 65 years and older. The 65 years and older category serves as the reference group. Sex categories entered into the model included male and female with the male category acting as the reference group. Race categories were entered into the model as follows: Black non-Hispanic, Hispanic, Other non-Hispanic (American Indian or Alaskan Native, Asian, Native Hawaiian, or other Pacific Islander), multiracial non-Hispanic, and non-Hispanic White (reference group). Level of education categories included did not graduate high school, graduated high school, attended college/technical school, and graduated from college/technical school (reference group). Marital status categories entered into the model included divorced, widowed, separated, never married, a member of an unmarried couple, married/partnered, and not married/partnered. Those who were married/partnered served as the reference group. Employment status was entered into the model as employed for wages, self-employed, out of work one year or more, out of work less than one year, homemaker, student, retired, or unable to work. Those who were employed for wages served as the reference group. Health insurance status categories were yes (have health insurance) and no (do not have health insurance), with those who had health insurance serving as the reference group. Lastly, household income level was entered into the model as earning less than $15,000, $15,000 to $24,999, $25,000 to $34,999, $35,000 to $49,999, and $50,000 or more which served as the reference group.

BRFSS study documentation including survey data, methodology, design, and guidelines for complex sampling weights are publicly available. IRB approval was sought, and the IRB determined that the proposed activity did not meet the definition of human subjects research and was not subject to oversight by the Institutional Review Board.

To account for the complex sampling design, variables were weighted in all models and the reported N was adjusted using weighted analysis per the BRFSS guidelines for complex sampling weights Participants who responded “don’t know” or “refused were excluded from the analysis.

Prevalence rates were reported as percentages and a *P* value was considered significant if it was < .05. SAS 9.4 was used to perform all statistical analyses.

## Results and discussion

### Demographics

As seen in Table 1, the proportion of participants in each age category of the weighted study population were as follows: 11% were between the ages of 18–24, 17% were between the ages of 25–34, 17% were between the ages of 35–44, 20% were between the ages of 45–54, 20% were between the ages of 55–64, and 16% were 65 years or older. In terms of sex and race, most participants were female (64%), 70% of respondents were White non-Hispanic, 10% were Black non-Hispanic, 13% were Hispanic, 4% were Other non-Hispanic, and 2% were Multiracial non-Hispanic. Around 36% of participants were employed for wages, 6% were self-employed, 6% were out of work for more than one year, 4% were out of work for less than one year, 7% were homemakers, 5% were students, 15% were retired, and 20% were unable to work. In terms of marital status, 41% of participants were married/partnered, 16% were divorced, 8% were widowed, 4% were separated, 25% were never married, and 5% were part of an unmarried couple. Around 18% of participants did not graduate from high school, 28% graduated high school, 33% attended some college or technical school, and 20% graduated college or technical school. Regarding annual household income, 18% of participants earned less than $15,000, 19% earned between $15,000 and $24,999, 9% earned $25,000 to $34,999, 11% earned $35,000 to $49,999, and 28% earned more than $50,000 per year. Most participants had health insurance (85%).

**Table 1:** Summary of demographics.

Characteristic	Frequency	Weighted Frequency	Row Percent
**Sex**			
Male	224,773	15,628,969	36.2
Female	396,084	27,540,662	63.79
**Age**			
18–24 years	68,798	4,783,673	11.08
25–34 years	104,128	7,240,271	16.77
35–44 years	101,210	7,037,354	16.32
45–54 years	121,328	8,436,190	19.54
55–64 years	120,210	8,358,476	19.35
>65 years	96,677	6,722,184	15.56
**Education**			
Did not graduate High School	113,255	7,874,928	18.24
Graduated High School	174,789	12,153,466	28.15
Attended College or Technical School	204,779	14,238,768	32.98
Graduated College or Technical School	126,233	8,777,264	20.33
**Race**			
White, Non-Hispanic	434,022	30,178,590	69.9
Black, Non-Hispanic	59,422	4,131,747	9.57
Other, Non-Hispanic	24,402	1,696,736	3.93
Multiracial, Non-Hispanic	12,667	880,749	2.04
Hispanic	81,216	5,647,152	13.08
**Marital Status**			
Married	256,440	17,830,841	41.30
Divorced	101,520	7,058,940	16.35
Widowed	46,941	3,263,950	7.56
Separated	26,762	1,860,797	4.31
Never married	153,305	10,659,648	24.69
A member of an unmarried couple	33,157	2,305,489	5.34
**Employed**			
Employed for wages	224,959	15,641,921	36.23
Self-employed	37,503	2,607,706	6.04
Out of work for more than 1 year	34,275	2,383,202	5.52
Out of work for less than 1 year	27,817	1,934,193	4.48
A homemaker	44,334	3,082,620	7.14
A student	30,798	2,141,428	4.96
Retired	91,523	6,363,840	14.74
Unable to work	126,295	8,781,581	20.34
**Health Insurance Status**			
Yes	527,222	36,658,999	84.91
No	91,337	6,350,888	14.71
**Income**			
<$15,000	111,765	7,771,311	18.00
$15,000 - $24,999	117,975	8,203,050	19.00
$25,000 - $34,999	58,677	4,079,938	9.45
$35,000 - $49,999	66,438	4,619,613	10.70
>$50,000	178,887	12,438,414	28.81

#### Model 1

Table 2 shows the adjusted odds ratio (AOR) for respondents who reported poor general health. Overall, the model does not indicate a statistically significant difference for those who rate their general health as fair or poor in early years of ACA implementation as compared to those in later years of implementation.

**Table 2:** Self-reported poor general health status.

Characteristic	AOR	95% CI	
**ACA**			
Early Years of Implementation	0.99	0.96	1.03
Later Years of Implementation	Reference	---	---
**Age**			
18–24	0.36*	0.31	0.42
25–34	0.43*	0.39	0.48
35–44	0.64*	0.59	0.70
45–54	0.88*	0.82	0.97
55–64	0.96*	0.88	0.93
65+	Reference	---	---
**Gender**			
Male	Reference	---	---
Female	1.17*	1.13	1.22
**Race/Ethnicity**			
White only, Non-Hispanic	Reference	---	---
Black only, Non-Hispanic	0.83*	0.78	0.89
Other race only, Non-Hispanic	1.22*	1.08	1.37
Multiracial, Non-Hispanic	1.20*	1.07	1.33
Hispanic	1.13*	1.06	1.21
**Education Level**			
Did not graduate High School	2.11*	1.97	2.25
Graduated High School	1.51*	1.43	1.60
Attended College or Technical School	1.39*	1.31	1.47
Graduated from College or Technical School	Reference	---	---
**Marital status**			
Married	Reference	---	---
Divorced	0.91*	0.86	0.96
Widowed	0.91*	0.86	0.97
Separated	1.00	0.91	1.09
Never married	0.73*	0.68	0.78
A member of an unmarried couple	0.80*	0.71	0.90
**Employment Status**			
Employed for wages	Reference	---	---
Self-employed	1.10	0.97	1.25
Out of work for more than 1 year	2.67*	2.42	2.94
Out of work for less than 1 year	1.67*	1.47	1.90
A homemaker	1.73*	1.56	1.93
A student	1.18	0.96	1.44
Retired	2.64*	2.44	2.87
Unable to work	8.57*	8.00	9.17
**Health Insurance status**			
Insured	Reference	---	---
Uninsured	1.25*	1.17	1.33
**Income ($)**			
Less than $15,000	2.52*	2.33	2.72
$15,000 to less than $24,999	1.95*	1.82	2.09
$25,000 to less than $34,999	1.63*	1.51	1.76
$35,000 to less than $49,999	1.36*	1.26	1.47
$50,000 or more	Reference	---	---

*Notes*: * A *P* value of < .05 was considered significant.

#### Model 2

Table 3 shows respondents who reported 14 or more days when their physical health was not good, including physical illness or injury. In early years of ACA implementation, respondents were less likely to report 14 or more days of poor physical health as compared to later years of the ACA (AOR  =  0.96; 95% CI: 0.95, 0.98) after adjusting for age, sex, race, education, marital status, employment status, health insurance status and income.

**Table 3:** Self-reported 14 or more poor physical health days in the previous 30 days.

Characteristic	AOR	95% CI	
**ACA**			
Early Years of Implementation	0.96*	0.95	0.98
Later Years of Implementation	Reference	---	---
**Age**			
18–24	0.48*	0.46	0.51
25–34	0.63*	0.61	0.65
35–44	0.82*	0.79	0.84
45–54	1.05*	1.03	1.08
55–64	1.02*	1.00	1.04
65+	Reference	---	---
**Gender**			
Male	Reference	---	---
Female	1.03*	1.01	1.04
**Race/Ethnicity**			
White only, Non-Hispanic	Reference	---	---
Black only, Non-Hispanic	0.89*	0.87	0.92
Other race only, Non-Hispanic	1.13*	1.09	1.17
Multiracial, Non-Hispanic	1.30*	1.25	1.35
Hispanic	1.03*	1.00	1.05
**Education Level**			
Did not graduate High School	1.49*	1.45	1.53
Graduated High School	1.32*	1.30	1.35
Attended College or Technical School	1.35*	1.32	1.37
Graduated from College or Technical School	Reference	---	---
**Marital status**			
Married	Reference	---	---
Divorced	0.94*	0.92	0.96
Widowed	0.87*	0.85	0.89
Separated	1.04*	1.00	1.08
Never married	0.75*	0.73	0.76
A member of an unmarried couple	0.89*	0.86	0.93
**Employment Status**			
Employed for wages	Reference	---	---
Self-employed	1.11*	1.08	1.15
Out of work for more than 1 year	2.60*	2.52	2.69
Out of work for less than 1 year	1.78*	1.71	1.85
A homemaker	1.64*	1.59	1.69
A student	1.23*	1.16	1.31
Retired	2.16*	2.11	2.22
Unable to work	7.90*	7.73	8.08
**Health Insurance status**			
Insured	Reference	---	---
Uninsured	1.01	0.98	1.03
**Income ($)**			
Less than $15,000	2.23*	2.18	2.29
$15,000 to less than $24,999	1.91*	1.87	1.96
$25,000 to less than $34,999	1.56*	1.52	1.60
$35,000 to less than $49,999	1.35*	1.32	1.38
$50,000 or more	Reference	---	---

*Notes*: * A *P* value of < .05 is considered significant.

#### Model 3

Bad mental health days which included stress, depression, and problems with emotions for 14 or more days are shown in Table 4. In early years of ACA implementation, patients were less likely to have 14 or more days of poor mental health as compared to later years of implementation after adjusting for age, sex, race, education, marital status, employment status, health insurance status and income (AOR  =  0.93; 95% CI: 0.92, 0.94).

**Table 4:** Self-reported 14 or more poor mental health days in the previous 30 days.

Characteristic	AOR	95% CI	
**ACA**			
Early Years of Implementation	0.93*	0.92	0.94
Later Years of Implementation	Reference	---	---
**Age**			
18–24	2.14*	2.06	2.23
25–34	1.92*	1.87	1.98
35–44	1.84*	1.79	1.89
45–54	1.73*	1.69	1.77
55–64	1.39*	1.36	1.42
65+	Reference	---	---
**Gender**			
Male	Reference	---	---
Female	1.00	0.98	1.01
**Race/Ethnicity**			
White only, Non-Hispanic	Reference	---	---
Black only, Non-Hispanic	1.01	0.98	1.03
Other race only, Non-Hispanic	1.12*	1.08	1.16
Multiracial, Non-Hispanic	1.23	1.19	1.28
Hispanic	0.98	0.96	1.01
**Education Level**			
Did not graduate High School	1.39*	1.36	1.42
Graduated High School	1.34*	1.31	1.36
Attended College or Technical School	1.30*	1.28	1.33
Graduated from College or Technical School	Reference	---	---
**Marital status**			
Married	Reference	---	---
Divorced	1.10*	1.08	1.12
Widowed	1.03*	1.01	1.05
Separated	1.38*	1.33	1.42
Never married	0.99	0.97	1.01
A member of an unmarried couple	0.99	0.96	1.02
**Employment Status**			
Employed for wages	Reference	---	---
Self-employed	0.98	0.96	1.01
Out of work for more than 1 year	2.09*	2.03	2.15
Out of work for less than 1 year	1.79*	1.73	1.85
A homemaker	1.20*	1.17	1.24
A student	1.14*	1.10	1.19
Retired	1.32*	1.29	1.35
Unable to work	3.19*	3.13	3.25
**Health Insurance status**			
Insured	Reference	---	---
Uninsured	1.19*	1.17	1.22
**Income ($)**			
Less than $15,000	1.91*	1.87	1.95
$15,000 to less than $24,999	1.67*	1.64	1.71
$25,000 to less than $34,999	1.42*	1.39	1.46
$35,000 to less than $49,999	1.30*	1.27	1.33
$50,000 or more	Reference	---	---

*Notes*: * A *P* value of < .05 is considered significant.

#### Model 4

Table 5 shows respondents who reported 14 or more days of overall poor health including days that their physical or mental health kept them from doing their usual activities, such as self-care, work, or recreational activities. Those in the early years of ACA implementation were less likely than their counterparts in later years of ACA implementation to report 14 or more days of overall poor physical and mental health (AOR  =  0.93; 95% CI: 0.90, 0.96).

**Table 5:** Self-reported 14 or more poor physical and mental days including activity limited days in the previous 30 days.

Characteristic	AOR	95% CI	
**ACA**			
Early Years of Implementation	0.93*	0.90	0.96
Later Years of Implementation	Reference	---	---
**Age**			
18–24	0.98	0.89	1.09
25–34	1.15*	1.07	1.23
35–44	1.34*	1.26	1.43
45–54	1.49*	1.40	1.57
55–64	1.22*	1.16	1.28
65+	Reference	---	---
**Gender**			
Male	Reference	---	---
Female	1.12*	1.08	1.16
**Race/Ethnicity**			
White only, Non-Hispanic	Reference	---	---
Black only, Non-Hispanic	0.99	0.93	1.05
Other race only, Non-Hispanic	1.16*	1.04	1.29
Multiracial, Non-Hispanic	1.21*	1.10	1.33
Hispanic	0.99	0.94	1.05
**Education Level**			
Did not graduate High School	1.45*	1.37	1.54
Graduated High School	1.33*	1.28	1.39
Attended College or Technical School	1.33*	1.28	1.39
Graduated from College or Technical School	Reference	---	---
**Marital status**			
Married	Reference	---	---
Divorced	1.03	0.98	1.07
Widowed	0.95	0.90	1.01
Separated	1.04	0.96	1.12
Never married	0.85*	0.80	0.90
A member of an unmarried couple	0.90*	0.82	0.98
**Employment Status**			
Employed for wages	Reference	---	---
Self-employed	1.50*	1.37	1.63
Out of work for more than 1 year	4.62*	4.31	4.96
Out of work for less than 1 year	3.13*	2.90	3.39
A homemaker	2.21*	2.05	2.37
A student	1.62*	1.44	1.82
Retired	3.70*	3.48	3.93
Unable to work	10.96*	10.42	11.52
**Health Insurance status**			
Insured	Reference	---	---
Uninsured	1.07*	1.02	1.13
**Income ($)**			
Less than $15,000	1.86*	1.75	1.98
$15,000 to less than $24,999	1.53*	1.45	1.61
$25,000 to less than $34,999	1.37*	1.29	1.45
$35,000 to less than $49,999	1.27*	1.20	1.34
$50,000 or more	Reference	---	---

*Notes*: * A *P* value of < .05 is considered significant.

According to the main effects of the regression models, those individuals with DD in early years of ACA implementation were less likely to report 14 or more days of poor physical health, less likely to report 14 or more days of poor mental health, and less likely to report 14 or more days of overall poor physical and mental health as opposed to later years of ACA implementation. Although outcome data is still forthcoming, our results are consistent with some studies that have found poorer outcomes for those with mental health illnesses following ACA implementation [22].

A reason for our findings could be that this study focused on the United States as a whole. The United States has long suffered from a fragmented health care system especially as it pertains to mental health. Given that the ACA was a federal policy with many provisions that went into effect at the national level, other provisions such as Medicaid expansion were left up to the states to implement. Only 39 states including Washington DC have Medicaid expansion coverage [23]. An additional 12 states have not adopted Medicaid expansion [23]. Medicaid is the largest payer for mental health services and provides coverage for over 25% of those adults with serious mental illness [14, 24]. In many cases, Medicaid mental health coverage is more comprehensive than benefits provided by private insurers [25]. Studies that have focused on Medicaid expansion states alone have found that patients have a reduction in psychological stress and have better self-reported physical health, mental health, and HRQoL [26, 27]. By examining the U.S. as a whole, mental health benefits that are seen under Medicaid expansion in later years of the ACA may not be as obvious when including those states that have chosen not to participate in Medicaid expansion. This underscores the need and the importance for the federal government to take a larger more comprehensive role coordinating and evaluating changes [28] within the health care system and implementing changes on a federal level.

Another reason for our results could be that although mental health benefits have been expanded under the ACA, those benefits do not always translate into improved access or improved patient reported outcomes. Worsening provider access issues may account for why patients with DD have a decrease in HRQoL in later years of the ACA. It is estimated that roughly half of patients with mental health issues are treated by primary care physicians (PCP) [29]. Mental health professions face national workforce shortages [30]. These shortages have been progressively getting worse not only in terms of the sheer number of mental health providers, but also in the geographic distribution of providers. Rural and underserved areas are known to have a dearth of mental health professionals [29, 30]. Depression can worsen and a reduction in HRQoL can occur if depression is left untreated or inappropriately managed. It has been reported that primary care providers have not been sufficiently trained in mental health and this can result in inadequate therapy (not prescribing enough medication), excessive therapy (prescribing too much medication), or ineffective treatment (not prescribing the proper medication) [31]. Research has shown that PCPs fail to provide adequate information on drug side effects and as a result, patients stop taking their medication as prescribed [31]. Moreover, mental health providers are known to more adequately explain common prescription side effects and the importance of medication adherence. Also, medication and psychotherapy, when used together, have been shown to be more successful in treating depression than medication alone [32]. Therefore, even if a primary care physician accurately prescribes medication, medication alone is frequently insufficient for appropriate care. Mental health providers are trained and able to manage, or coordinate, both drug and therapy aspects of a patient’s treatment.

Unfortunately, access to providers is complicated by the fact that many mental health professionals in private practice only accept a limited number of health plans if they accept health insurance at all [33, 34]. Often times patients with insurance will still have to pay full out of pocket expenses despite seeing an in-network provider. A small number of insurance plans offer out of network coverage; however, seeing a provider out of network results in high out of pocket expenses [35]. The financial burden of seeing a provider is a well-known deterrent to seeking mental health services [36]. Additionally, it has been reported that higher levels of financial stress and financial burden were linked to worse HRQoL and poor health perception [37]. The financial barriers faced by those with mental illness when seeking treatment from a mental health provider creates the need forpatients to seek treatment at a primary care provider. As previously discussed, treatment from a PCP may not lead to optimal care. In turn, both the lack of access to a mental health provider or financial burden could potentially account for the reduction in HRQoL as seen in this research.

There are limitations to this study. The BRFSS is a cross sectional survey that relies on self-reports. Cause and effect relationships cannot be identified. The BRFSS is a large population-based study and does not include separate questions for minor and major depressive disorders or questions specific to mental illness such as medication usage or questions that go into depth regarding depression and treatment. Additionally, the BRFSS has a singular question regarding history of depression. Research studies based on a singular criterion may lead to the inclusion of inappropriate study participants; however, in this instance, the use of a singular criterion from the BRFSS is known to be reliable and valid [38]. Despite these limitations, surveillance surveys such as the BRFSS play an important role in providing reliable and valid national estimates for HRQoL and a chronic disease such as DD [38].Additionally, although the main point of this research was to examine HRQoL for those with depressive disorders in early years of ACA implementation as compared to later years of the ACA, there are other interesting findings. Primarily added to the models as control variables, these variables in the models show that many disparities exist in HRQoL for those in racial and ethnic minority groups and for those with low socioeconomic status. Future studies should explicitly examine racial and ethnic disparities in HRQoL for those with DD that still exist despite the ACA.

## Conclusions

This research highlights areas for improvement. The persistent regulatory wrangling regarding the Affordable Care Act has led to less-than-optimal enrollments in health insurance plans and less than optimal access to much needed affordable health care services. Despite the incentives for Medicaid expansion, many states have not expanded their Medicaid programs. For those with mental illness who are uninsured, Medicaid expansion would increase access, potentially reduce costs, increase health care utilization for not only quality general health services, but to quality mental health services and could potentially improve patient outcomes. Furthermore, the federal government needs to address the mental health workforce shortage and the uneven geographic distribution of providers in order to strengthen health care access and health care service options for patients. Policy makers at all levels must be committed to a comprehensive approach for access to health services and mental health treatment.

## Supporting information

S1 Appendix (DOCX)
