# Supplementary material for: Impact of ACA implementation on health related quality of life among those with depressive disorders in the United States: A secondary data analysis of the 2011-2017 BRFSS
Source: PLoS One. 2022 Mar 31;17(3):e0266402. doi: 10.1371/journal.pone.0266402 (PMC8970490; doi:10.1371/journal.pone.0266402)
Supplement: S1 Appendix — (DOCX) [file pone.0266402.s001.docx]

**Appendix A**

**Measures of HRQoL**

Patient-reported outcomes (PRO) have become an important part of assessing health care throughout the world [1].  Health related quality of life is one type of PRO [1].  There are several measures used to assess HRQOL as well as related concepts of functional status including generic, as well as, disease-specific HRQoL measures.  These measures are multidimensional and focus on several different domains depending on the instrument [1].  The EQ-5D is the world's most extensively used instrument [2].  The EQ-5D is a generic measure as is the CDC HRQoL-4.  However, the EQ-5D has five dimensions (mobility, self, care, usual activities, pain/discomfort, anxiety/depression [2] whereas the CDC HRQoL-4 has four dimensions (self-rated general health, physical health, mental health, and activity limitations) [3]. The CDC measures HRQoL in the BRFFS and each item of the HRQOL-4 assesses a distinct domain that is thematically related [4]. For population health surveillance, the CDC HRQOL-4 has been shown to be valid and reliable in a variety of populations and health conditions such as chronic diseases, disability, risky health behaviors as well as morbidity, health care usage, and mortality [3].

1. Quittner, A. L., Nicolais, C. J., & Saez-Flores, E. (2018, March 13). *Integrating patient-reported outcomes into research and clinical practice*. Kendig's Disorders of the Respiratory Tract in Children (Ninth Edition). Retrieved January 12, 2022, from https://www.sciencedirect.com/science/article/pii/B9780323448871000134
2. Gusi N., Olivares P.R., Rajendram R. (2010) The EQ-5D Health-Related Quality of Life Questionnaire. In: Preedy V.R., Watson R.R. (eds) Handbook of Disease Burdens and Quality of Life Measures. Springer, New York, NY. https://doi.org/10.1007/978-0-387-78665-0_5
3. Centers for Disease Control and Prevention. (2000). Measuring Healthy Days Population Assessment of Health-Related Quality of Life. Retrieved from https://www.cdc.gov/hrqol/pdfs/mhd.pdf
4. Healthy People*.Related quality of life and well-being*. Health. (n.d.). Retrieved January 12, 2022, from https://www.healthypeople.gov/2020/about/foundation-health-measures/Health-Related-Quality-of-Life-and-Well-Being
